# Chemical insights into the synthetic chemistry of five-membered saturated heterocycles—a transition metal–catalyzed approach

**DOI:** 10.3389/fchem.2023.1185669

**Published:** 2023-07-26

**Authors:** Sunbal, Muhammad Alamzeb, Muhammad Omer, Obaid-Ur-Rahman Abid, Mohib Ullah, Muhammad Sohail, Ihsan Ullah

**Affiliations:** ^1^ Institute of Chemical Sciences, University of Swat, Swat, Pakistan; ^2^ Department of Chemistry, University of Kotli, Kotli, Pakistan; ^3^ Department of Chemistry, Hazara University, Mansehra, Pakistan; ^4^ Department of Chemistry, Balochistan University of Information Technology Engineering and Management Sciences (BUITEMS), Quetta, Pakistan

**Keywords:** heterocycles, saturated, five-membered, transition metals, synthesis, catalysis, green

## Abstract

Drug design and delivery is primarily based on the hunt for new potent drug candidates and novel synthetic techniques. Recently, saturated heterocycles have gained enormous attention in medicinal chemistry as evidenced by the medicinal drugs listed in the FDA Orange Book. Therefore, the demand for novel saturated heterocyclic syntheses has increased tremendously. Transition metal (TM)–catalyzed reactions have remained the prime priority in heterocyclic syntheses for the last three decades. Nowadays, TM catalysis is well adorned by combining it with other techniques such as bio- and/or enzyme-catalyzed reactions, organocatalysis, or using two different metals in a single catalysis. This review highlights the recent developments of the transition metal–catalyzed synthesis of five-membered saturated heterocycles.

## 1 Introduction

Regarding synthetic chemistry, recent decades have witnessed spectacular progress in transition metal–catalyzed reactions (Chakraborty et al., 2022). In recent years, considerable progress has been achieved in the improvement of building block availability, reaction conditions, ligands, and metals, making this method one of the most powerful tools for both academic and industrial researchers. These entities provide effective control over the regio- and stereoselectivity of complicated reactions, enabling novel bond formation, which can only be achieved using catalysis (Skubi et al., 2016; Ullah et al., 2022). Through tremendous research efforts, scientists have achieved exceptional success in the understanding of metal properties and the utilization of their diverse reactivities in different organic transformations; however, much remains to be explored (Ali et al., 2013). Most recently, very effective attempts have been made by combining pairs of different catalytic patterns in divergent synthetic modifications, including the combination of different transition metals in single catalysis, the combination of transition metal catalysis with enzyme and/or bio-catalysis, and the combination of transition metal catalysis with organocatalysis (Ullah et al., 2011; Kim et al., 2020). On the other hand, heterocycles are of immense importance, and therefore, scientists are making exhaustive efforts to synthesize these heterocyclic compounds through novel, cost-effective, and green synthetic transformations (Ibad et al., 2010). Among all the available synthetic techniques, transition metal–catalyzed reactions are the most popular for the synthesis of heterocyclic compounds due to the fact that complicated molecules can be easily synthesized from readily available starting materials under mild reaction conditions (Ullah et al., 2010a; Khera et al., 2010; Tang et al., 2023b).

Heterocycles—a major division among metabolites and synthetic compounds—constitute a major portion (85%) of physiologically active compounds (Kabir and Uzzaman, 2022). This affirms the importance of heterocycles during drug design in medicinal chemistry (Ullah et al., 2022). Heterocycles are present in the majority of naturally occurring compounds, including nucleic acids (DNA and RNA), carbohydrates (ring forms), hemoglobin, chlorophyll, vitamins, etc. Heterocycles are also constituents of proteins, antibiotics, hormones, and vitamins. Amino acids, such as histidine, tryptophan, and proline, as well as vitamins and synthetase precursors such as pyridoxine, riboflavin, thiamine, biotin, folic acid, and B12 are some of the most prevalent heterocycles (Ali et al., 2009; Ullah et al., 2009). Not only are they part of the structural units of living organisms, they impart a huge array of biological and medicinal properties, including, but not limited to, antibacterial, antiviral, antifungal, anti-inflammatory, anti-oxidant, anti-HIV, anti-cancer, antidiabetic, antidepressant, anti-microbial, anti-malarial, anti-neoplastic, anti-tubercular, anti-histaminic, anticoagulant, anti-allergic, enzyme inhibitors, anesthetics, and immunomodulatory agents (Nawaz et al., 2011; Nawaz et al., 2012). Further, they are also prominent in agrochemicals, the food industry (coloring and flavoring agents), copolymers, photostabilizers, fluorescents, and corrosion protection chemicals (Langer et al., 2011; Song et al., 2022).

The importance of saturated heterocycles is well established in medicinal chemistry, as evidenced from the medicinal drugs listed in the FDA Orange Book (Taylor et al., 2014). The majority of biologically active compounds contain between one and four rings (Taylor et al., 2014), partially due to their lower conformational entropy, enabling them to bind to the target site. In addition, saturated rings are intrinsically more drug-like than their planar aromatic counterparts, with the advantages of better water solubility and the avoidance of arene oxidation, unlike aromatic rings, while imparting better three-dimensional occupancy of the target drug (Mitrofanov and Beletskaya, 2023). These factors contribute favorably toward the recent criteria for drug-likeness formulations, i.e., a higher degree of saturation and a chiral center (Ullah et al., 2010b; Marson, 2017). A large number of successful clinical trials have revealed that the most suitable ratio of sp^3^ carbons to the total number of carbons per molecule having at least one asymmetric center is 0.47 (Betschart et al., 2013; Tang and Song, 2023). While avoiding the usage of rings that are entirely heteroaromatic, such as that in furan, pyridine and imidazole have additional advantages, including a higher structural diversity (owing to stereoisomerism) for a small increase in molar mass. As a result, the structure of drug-like candidates is changing (Bauer et al., 2021), with more emphasis placed on saturated rings such as heterocycles (Ahmad Khera et al., 2010). Further, the combination of saturated heterocycles with aromatic substituents caters for special, attractive screening of sole compounds or lead-like compounds due to the different hidden available binding interactions (Croft et al., 2019; Tang et al., 2023a).

**FIGURE 1:**
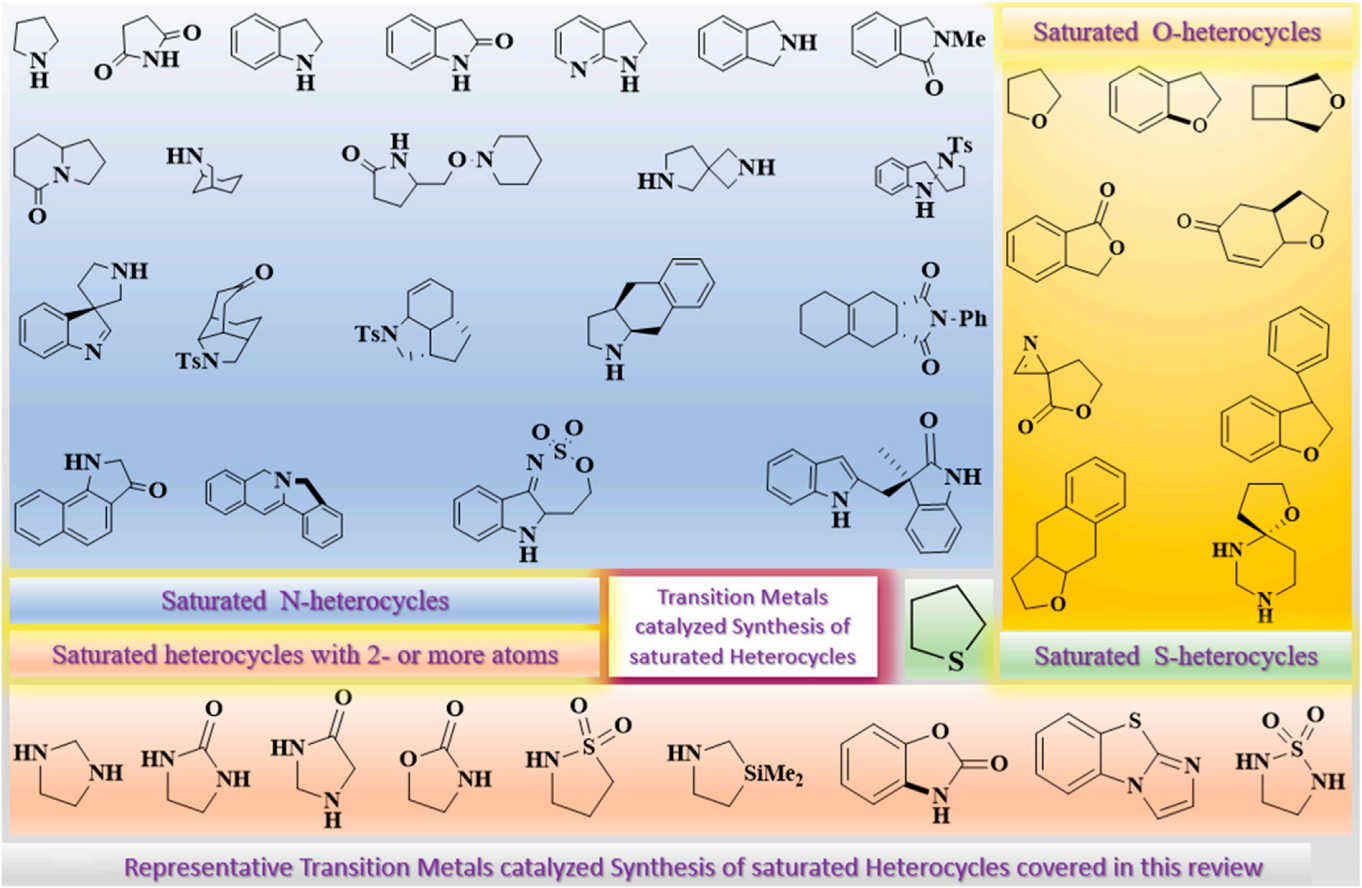
Representative Transition Metals catalyzed Synthesis of saturated Heterocycles covered in this review.

This review briefly and concisely summarizes the recent developments toward the synthesis of five-membered saturated heterocycles through transition metal–catalyzed reactions. All five-membered saturated N-, O-, and S-heterocycles that are either monocyclic, bicyclic, benzofused, or multicyclic, obtained from any substrate, have been covered, as shown in Figure 1. Further, reaction schemes, specific reactions conditions, etc., have been shown alongside, which are mandatory. All types of reactions, including multicomponent and multistep reactions, have been summarized.

**FIGURE 2:**
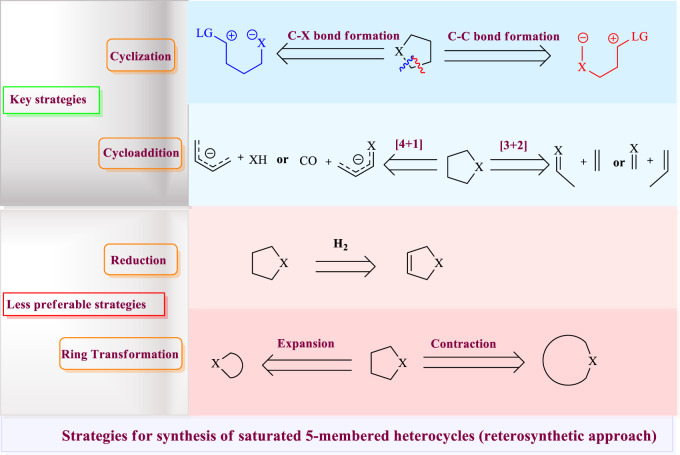
Strategies for synthesis of saturated 5-membered heterocycles (reterosynthetic approach).

A comprehensive literature survey for the preparation of saturated heterocycles by transition metal–catalyzed reactions was carried out. It was found that four (4) strategies are generally used: **1)** cyclization through carbon–heteroatom bond formation or carbon–carbon bond formation, **2)** [3 + 2] or [4 + 1] cycloaddition, **3)** reduction of unsaturated five-membered rings, and **4)** ring transformation either through ring expansion or contraction, as shown in Figure 2. These methods bear distinct strategic considerations and offer various opportunities and limitations. The first two methods are the most commonly applied and key strategies viz. cyclization and cycloaddition. Cycloadditions are generally more responsive to confluent syntheses in comparison to cyclizations, having the advantage of an intermolecular nature. Although cyclizations commonly generate stoichiometric waste, they may require protecting group usage, leading to longer syntheses; however, a variety of delicate cyclization approaches have been developed, making this straightforward strategy largely successful and commonly used. In rare cases, reduction is also applied to the commonly available unsaturated five-membered ring to obtain saturated five-membered heterocycles. Due to the widespread preparative methods of four- and six-membered heterocycles, ring expansion and contraction approaches from available precursors provide attractive routes to 5-membered heterocycles.

**FIGURE 3:**
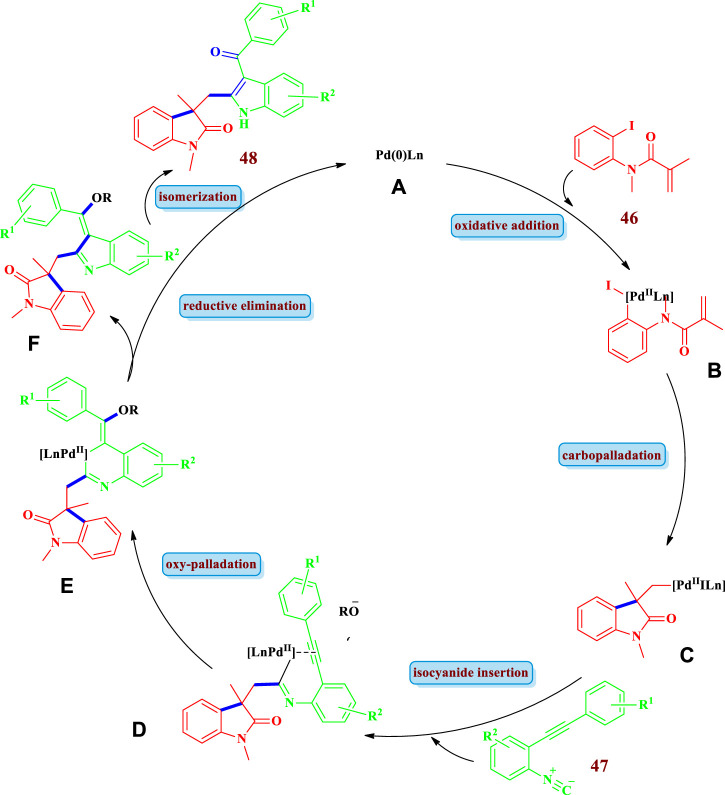
Representative mechanism for Transition metal catalyzed saturated heterocycles synthesis (Proposed for Scheme-4G) catalayzed by Palladium (Chen et al., 2021)

**SCHEME 1:**
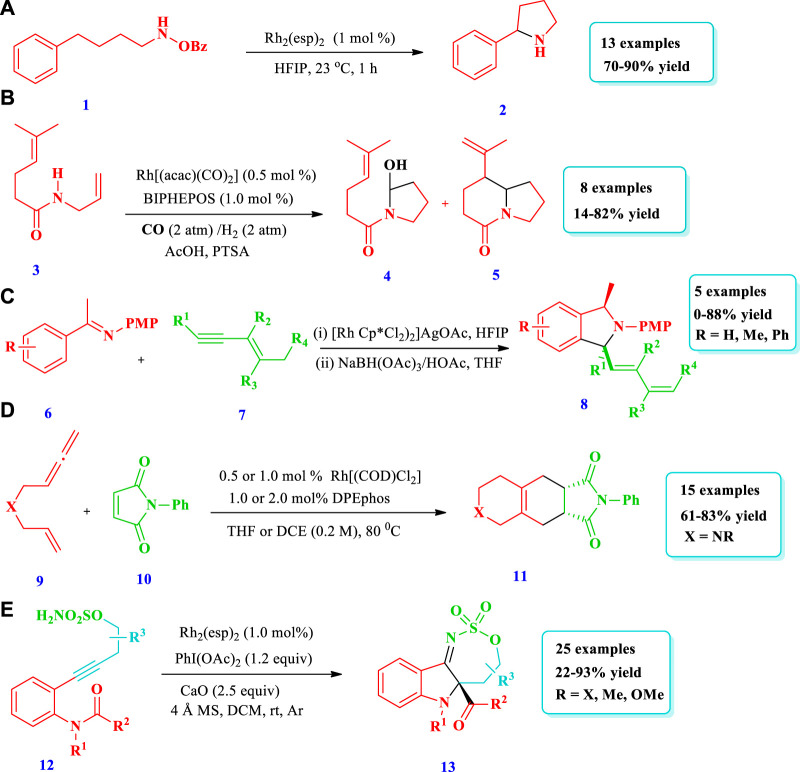
Rh catalyzed synthesis of saturated N-heterocycles.

**SCHEME 2:**
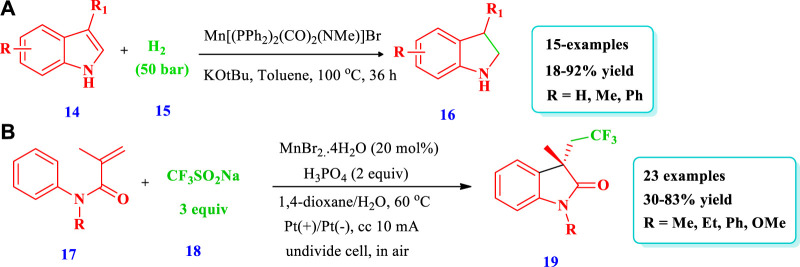
Mn catalyzed synthesis of saturated N-heterocycles.

**SCHEME 3:**
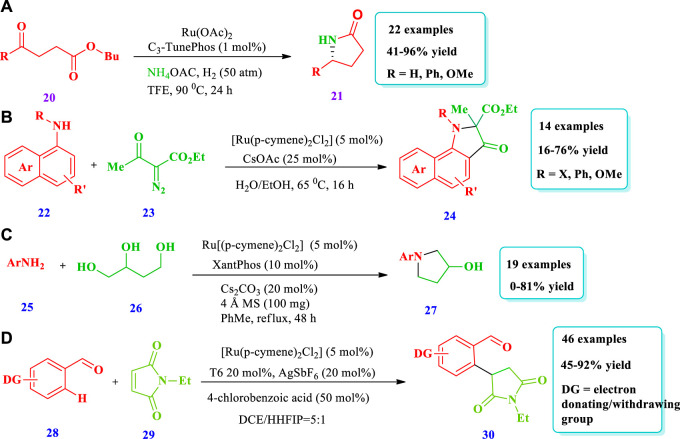
Ru catalyzed synthesis of saturated N-heterocycles.

**SCHEME 4:**
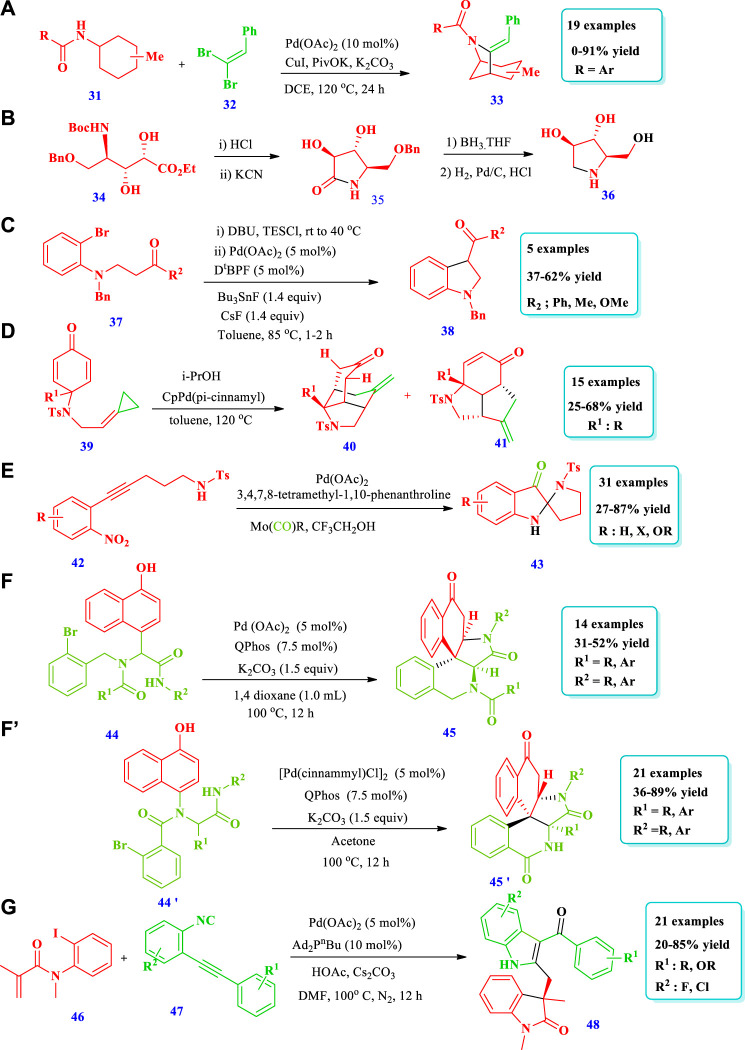
Palladium catalyzed synthesis of saturated N-heterocycles.

**SCHEME 5:**
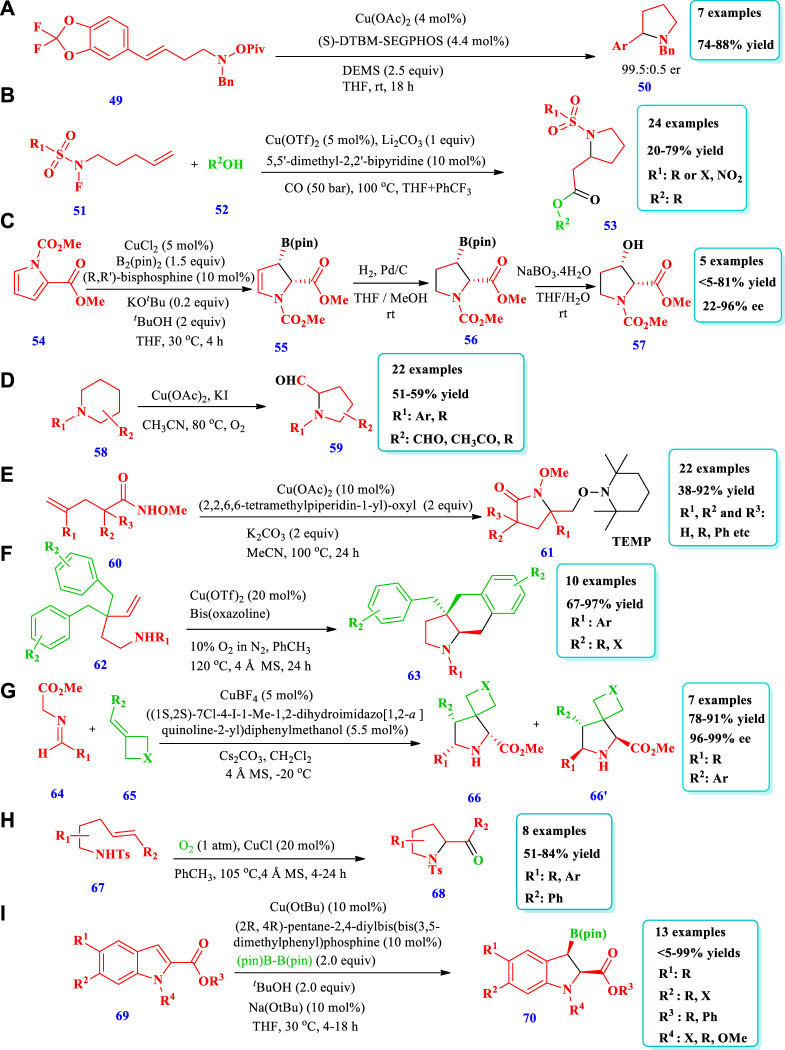
Copper catalyzed synthesis of saturated N-heterocycles.

**SCHEME 6:**
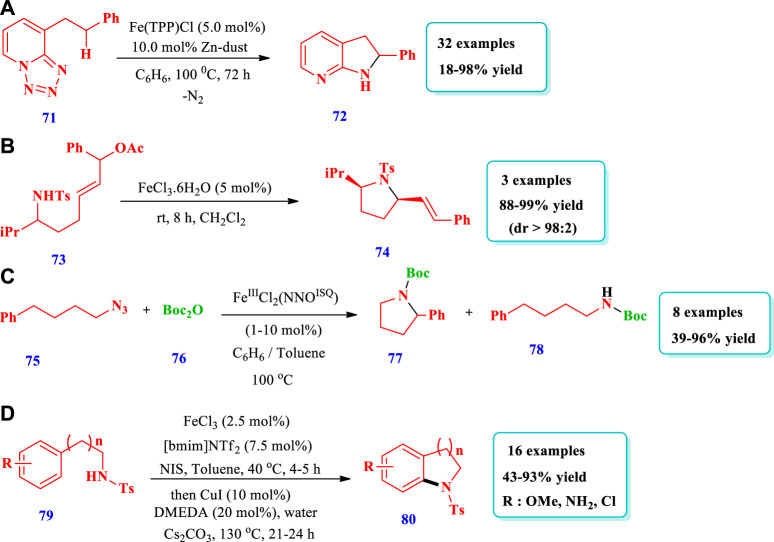
Fe catalyzed synthesis of saturated N-heterocycles.

**SCHEME 7:**
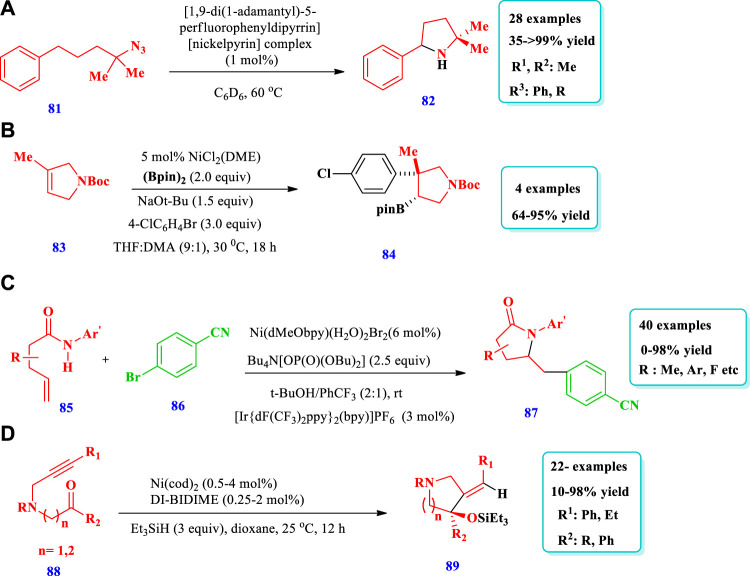
Ni catalyzed synthesis of saturated N-heterocycles.

**SCHEME 8:**
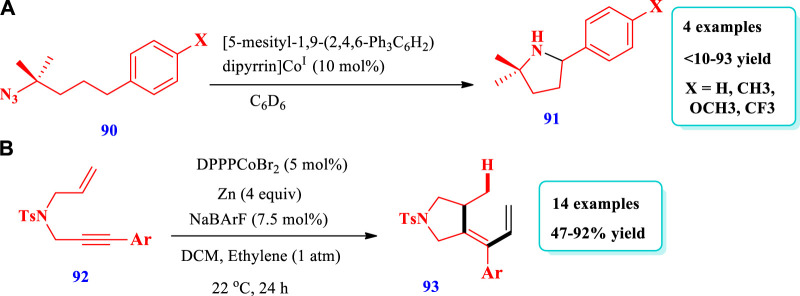
Cobalt catalyzed synthesis of saturated N-heterocycles.

**SCHEME 9:**
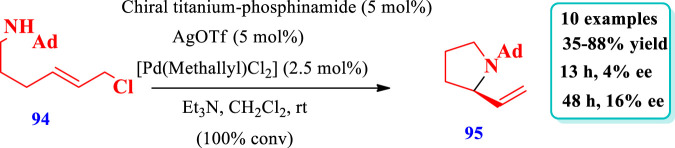
Ti/Pd complex catalyzed synthesis of saturated N-heterocycles.

**SCHEME 10:**
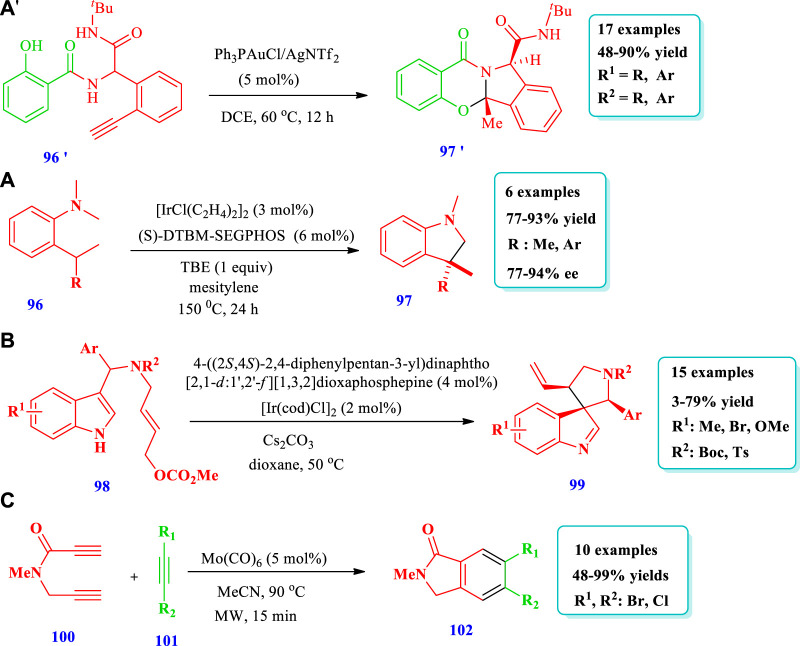
Gold and Irridium catalyzed synthesis of saturated N-heterocycles.

**SCHEME 11:**
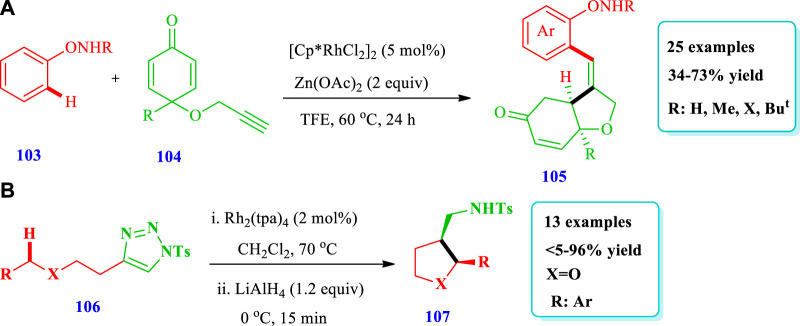
Rh catalyzed synthesis of saturated O-heterocycles.

**SCHEME 12:**
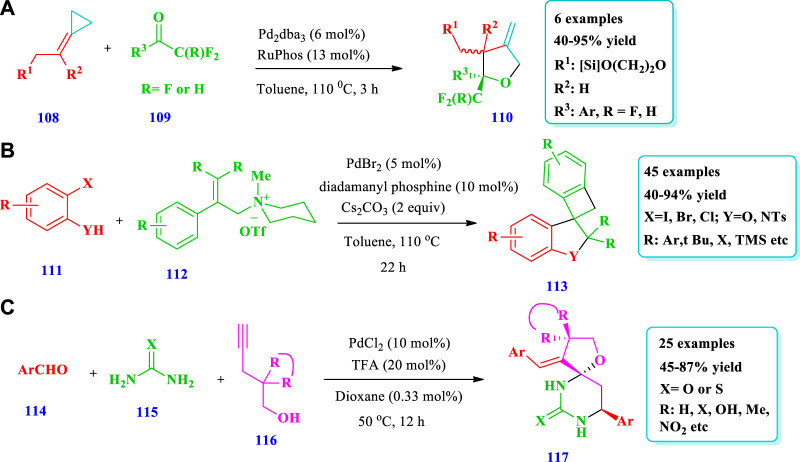
Pd catalyzed synthesis of saturated O-heterocycles.

**SCHEME 13:**
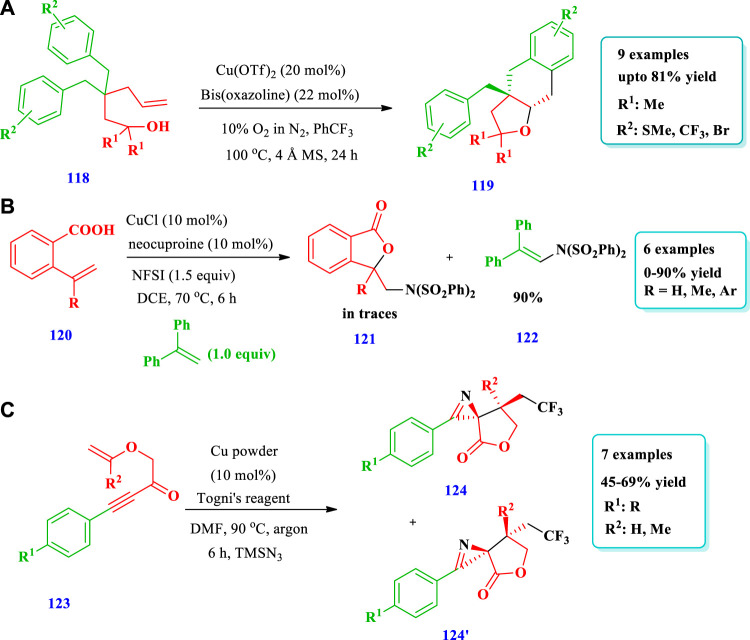
Cu catalyzed synthesis of saturated O-heterocycles.

**SCHEME 14:**
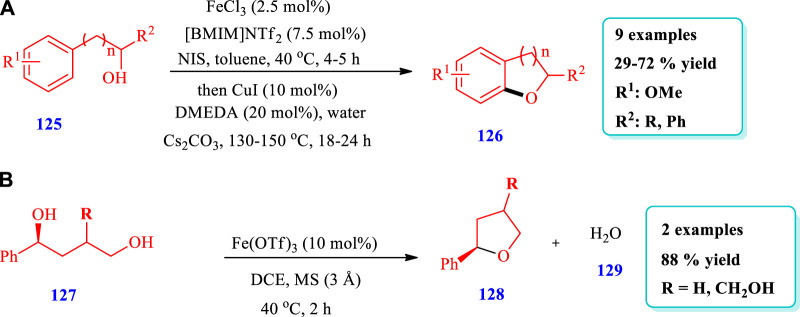
Fe catalyzed synthesis of saturated O-heterocycles.

**SCHEME 15:**
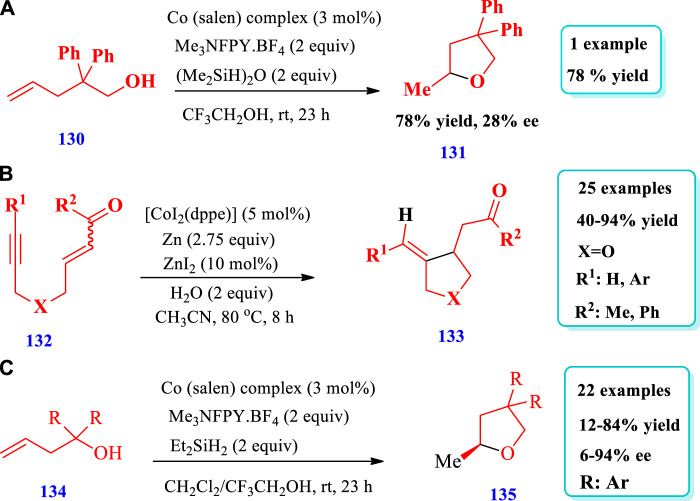
Co catalyzed synthesis of saturated O-heterocycles.

**SCHEME 16:**
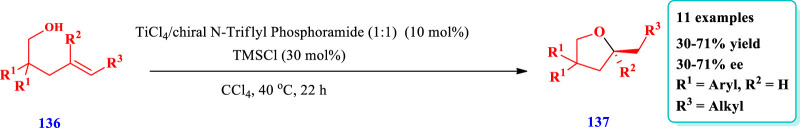
Ti catalyzed synthesis of saturated O-heterocycles.

**SCHEME 17:**
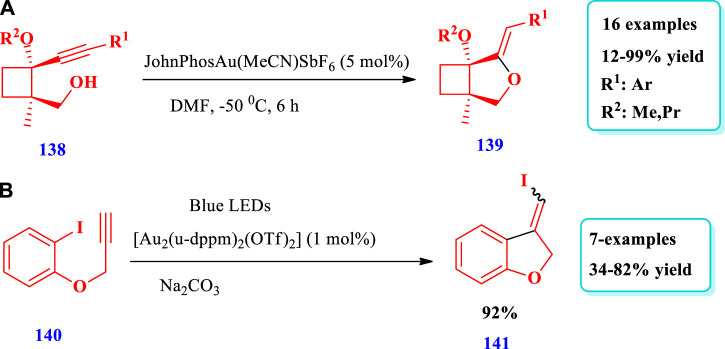
Au catalyzed synthesis of saturated O-heterocycles.

**SCHEME 18:**
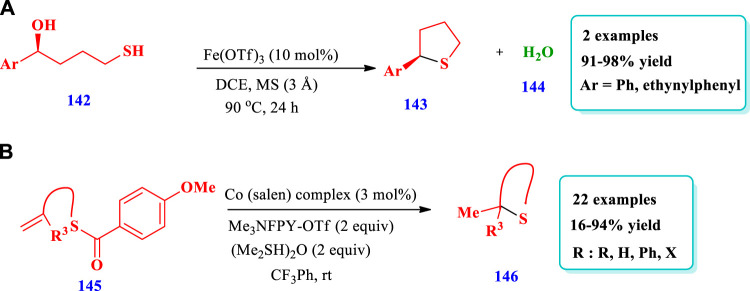
Fe and Co catalyzed synthesis of saturated O-heterocycles.

**SCHEME 19:**
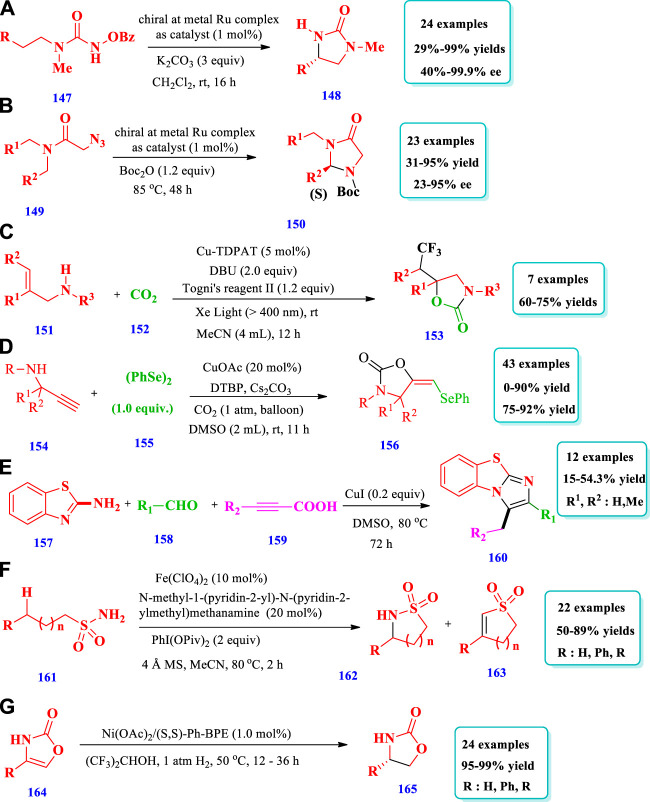
Transition metal catalyzed synthesis of saturated heterocycles with two or more atoms.

**SCHEME 20:**
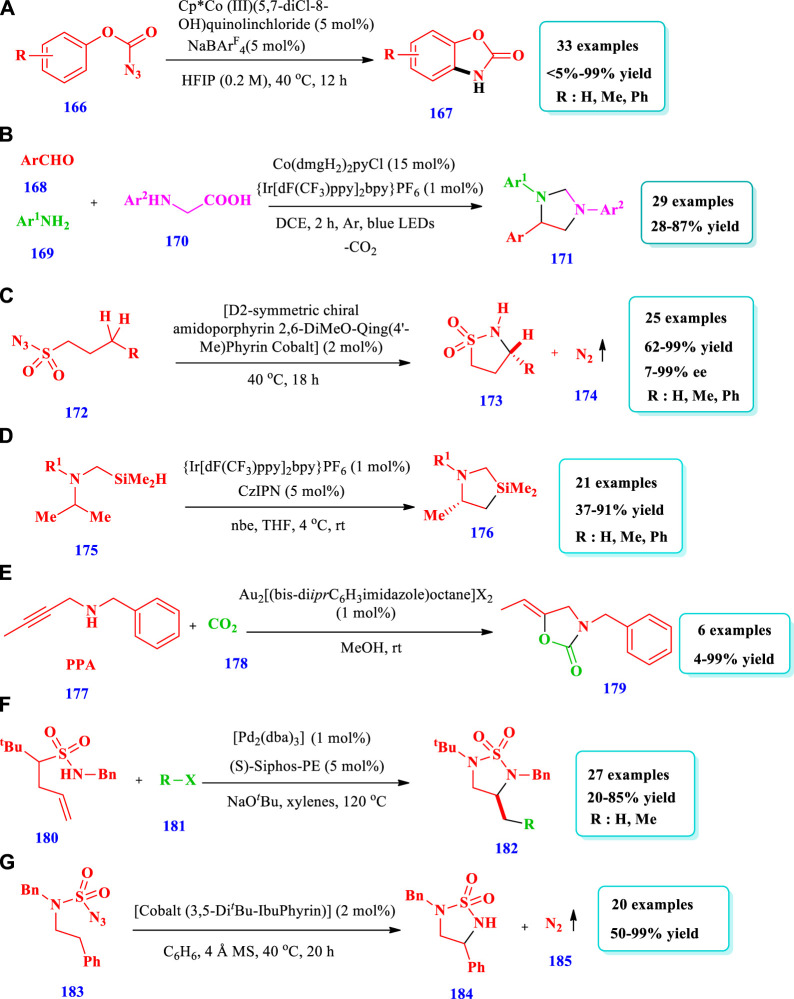
Transition metal catalyzed synthesis of saturated heterocycles with two or more atoms.

Regarding the mechanism of reactions that are catalyzed by transition metals (or their complexes, etc.), they proceed through a chain of reactions in a cycle generally called the catalytic cycle. For the sake of better understanding and simplicity, this catalytic cycle can be divided into three main mechanistic steps: **1)** oxidative addition of the catalyst (transition metal) to the substrate, **2)** transmetallation (insertion of second substrate), and finally **3)** reductive elimination with the formation of the product and the regeneration of the catalyst. These steps are followed, be it in cyclization, cycloaddition, or any other method. However, depending on the nature of the reactants involved and the conditions applied, the mechanism can be elaborated and extended to more steps. This same pattern is also followed in the synthesis of saturated heterocycles catalyzed by transition metals. A representative catalytic cycle for Scheme-4G (Chen et al., 2021) catalyzed by palladium is shown in Figure 3. The reaction starts with the oxidative addition of Pd(0) to the C-I bond of the substrate; *N*-(2- Iodophenyl)-*N*-methylacrylamide **46** between the C to halogen bond, forming the arylpalladium species **A**, which undergoes intramolecular carbopalladation to form intermediate **B**. Subsequently, migratory insertion of 2-(2-phenylethynyl)phenyl isocyanide **47** into the intermediate **B** gives the imidoyl palladium species **C**. Intramolecular addition to the triple bond and further attack of an oxy-nucleophile forms intermediate **D**. Then, reductive elimination of intermediate **D** results in the formation of intermediate **E**, followed by an isomerization step to give the desired product **48**. The readers are encouraged to consult the specific corresponding references if interested in the detailed reaction mechanisms and/or specific catalyst systems and ligands involved in the specific scheme as complete details of the aforementioned is beyond the scope of this manuscript. The following sections (Schemes 1–20) of this manuscript will rather present a variety of saturated heterocycles (Figure 1) prepared by transition metal–catalyzed reactions using different strategies (Figure 2), mainly following the representative reaction mechanism (Figure 3).

With this review, we desire to achieve the following goals: primarily, the intent is to help and guide chemists interested in the synthesis of saturated heterocycles, by focusing on a diversified synthetic approach (Schemes 1–20). The majority of the syntheses are based on the key strategies of cyclization and cycloaddition (Figure 2). As a result, we hope to significantly reduce the time that is required to sift through a large dataset of literature on heterocycle synthesis. Second, we aim to highlight the exciting features and strategies with limited examples in this area, particularly with a focus on the modular nature that such an approach can bring to the table. Finally, the ideal goal is to accelerate and promote access to these effective heterocyclic motifs for therapeutic explorations and to discover life-saving medicines.

## 2 Saturated heterocycles containing one heteroatom

### 2.1 N-heterocycles

#### 2.1.1 Rhodium-catalyzed synthesis

Noda et al. (2020) explored a simple but very handy synthetic protocol catalyzed by rhodium to afford saturated N-heterocycles (Pyrrolidines, **2**) starting from O-benzoylhydroxylamines (**1**) (Noda et al. 2020). Without rhodium, the starting material **1** did not react at all; however, when rhodium was employed, excellent transformation occurred through C(sp^3^)-H functionalization. Overall, 13 different compounds have been synthesized by this reaction. The yields were good to excellent (70%–90%). When a 4-arylbutylamine derivative was used as the substrate, C-H insertion takes place at the benzylic position despite the position and electronic nature of the substituents on the benzene ring. In contrast, no (0%) yield was observed from heteroaryl-containing substrates (Scheme 1A). Similarly, Chiou et al. (2020) reported a domino hydroformylation double-cyclization method for the synthesis of pyrrolidine-fused azepane **(5)** via a Rh-catalyzed reaction from a trisubstituted alkene with an amide group **(3)** (Chiou et al., 2020). This strategy required mild reaction conditions for pyrrolidine-fused azepane, with good diastereoselectivity and yields (82%). Overall, eight (8) different compounds were synthesized by this reaction. During purification, mass loss of the monocyclized products **(4)** occurred due to the presence of acidic conditions for a long time, probably because of decomposition in silica gel. However, bicyclized products **(5)** were obtained in good yields (82%) when monocyclized crude residue was directly reacted with BF_3_OEt_2_ in DCM after evaporation for the removal of acetic acid (Scheme 1B). Bai et al. (2019) explored the synthetic protocol for saturated N-heterocycles **(8)** via the oxidative annulation of a benzene substrate **(6)** and 1,3-enynes **(7)** by sp^2^ and sp^3^ C-H activation catalyzed by Rh (III) in a two-step reaction (Bai et al. 2019). In the first step, an air sensitive intermediate (enamine) was produced. In the second step, the hydrogenation of intermediate takes place in order to facilitate product isolation and conversion into air stable products (amine), with 75% yield and low diastereoselectivity. This method required mild reaction conditions and allowed for a wide scope of substrates, high regioselectivity, and high efficiency. Different substituents such as halogens, electron withdrawing, and donating groups at the para-position of the phenyl group were tested by coupling with 1,3-enyne, giving moderate to good yield with >20:1 diastereoselectivity. The 1,3-enyne that was terminated with n-butyl or alkylamino groups did not react; however, 1,3-enyne with a trans butenyl group produced the best yield with high diastereoselectivity (Scheme 1C). Based on one-pot tandem Diels alder cycloaddition reactions of allenenes **(9)** with different nitrogen-bearing dienophiles **(10)**, Zhou Y. et al., 2019 reported various bicyclic and tricyclic N-heterocycles **(11)** catalyzed via rhodium/phosphine (Zhou Z. et al., 2019). This strategy was important because the desired products were synthesized with high chemo-, regio-, and diastereoselectivity under mild reaction conditions with 0.5 mol% catalyst loading and a wide scope for substrates. Fifteen (15) different compounds have been synthesized by this reaction. For complete conversion of the carbon-linked allenene substrate **(9)**, 1 mol% of catalyst loading was required, while for the protected amide allenenes, 0.5 mol% of catalyst loading was efficient (Scheme 1D). Hong et al. (2020) developed the synthesis of tricyclic 3-iminoindolines **(13)** through a rhodium-catalyzed nitrene/alkyne metathesis reaction of alkyne-tethered sufamates **(12)** (Hong et al. 2020). This strategy provides a broad substrate scope with excellent yields. The Rh-catalyzed nitrene/alkyne metathesis was based on the reaction intermediate α-imino metal carbene, followed by a carbene insertion reaction of the amide C-N bond using stevens [1,2]-acyl shift. Overall, 25 different compounds were prepared via this strategy, with moderate to excellent (22%–93%) yields (Scheme 1E).

#### 2.1.2 Manganese-catalyzed synthesis

Zubar et al. (2020) reported an efficient method to prepare saturated N-heterocycles (benzofuzed pyrrolidines **16**) via the hydrogenation of unsaturated N-heterocycles (indoles **14**) via a highly chemoselective manganese complex–catalyzed reaction (Zubar et al. 2020). In this method, hydrogen gas was used for reduction with the benefits of low cost and minimal waste production. Fifteen (15) different compounds have been synthesized by this reaction. The yield depends on the catalyst amount, solvent nature, and reaction time. The yield increased with an increase in the rection time and by using toluene as the solvent; however, the yield decreased with a decrease in the catalyst loading to 1 mol% and by using dioxane as the solvent (Scheme 2A). Zhang et al. (2019) developed the synthesis of azaheterocycles **(19)** via manganese-catalyzed electrochemical trifluoromethylation/C(sp^2^) functionalization in an oxidant-free environment using Langlois’ reagent as a CF_3_ source (Zhang et al., 2019). Overall, 23 different compounds have been synthesized by this reaction. Different substituents were well-tolerated and the yields were moderate to excellent (30%–83%) (Scheme 2B).

#### 2.1.3 Ruthenium-catalyzed synthesis

Shi et al. (2020) reported the Ru-catalyzed chiral NH Lactams **(21)** synthesis via asymmetric reductive amination/cyclization of keto acids/esters **(20)** (Shi et al., 2020). This methodology has the potential application of building enantioenriched lactams and benzo-lactams with high enantioselectivities (up to 97% ee) and excellent yields. The production of scalable and important drug intermediates further shows the importance of this strategy. Overall, 22 different compounds have been synthesized (Scheme 3A). Wang W. et al., 2020 reported the Ru-catalyzed synthesis of 2,2-disubstituted pi-extended 3-oxindoles **(24)** through the ortho-C-H alkylation reaction of napthylamines **(22)** with diazocompounds **(23)** (Wang X. et al., 2020). This strategy has the advantage of being green, using water as a solvent, and releasing benign N_2_ as a side-product. The fluorescent and antitumor properties of the product in water further expanded the importance of this strategy. Fourteen different compounds have been synthesized by this reaction. Good yields were obtained when DMSO or EtOH cosolvents were added in small quantities, improving the solubility of the substrate. However, using DMSO, EtOH, and DCE with the Ru-catalyst did not obtain the desired products. No product was obtained from electron-deficient N-phenyl-1-naphthylamine; however, alkyl substituent was tolerated and good yields were obtained (Scheme 3B). Xu et al. (2020) described a synthetic protocol to afford 3-pyrrolidinol **(27)** via the hydrogen auto-transfer amination cyclization of 1,2,4-butanetriol **(26)** with primary aromatic amines **(25)** using a ruthenium catalyst (Xu et al., 2020). With this strategy, various types of important 3-pyrrolidinol derivatives could be obtained from different types of substituted anilines. Nineteen (19) different compounds have been synthesized by this reaction. Good yields (81%) were obtained by using a CsCO_3_ base and a Xantphos ligand with [Ru(p-cymene) Cl_2_]_2_; the use of NaHCO_3_ and K_2_CO_3_ yielded no product. Moderate to good yields were obtained from meta or para-alkyl anilines; however, no yield (0%) was obtained from the reaction of triol with 2,6-dimethylaniline and 1-naphylamine due to steric effects. The phenolic-OH, due to its deactivation effect on the catalytic process, was inert for this reaction (Scheme 3C). Li et al. (2018) described the synthesis of pyrrolidines **(30)**, mediated by Ru ortho-C(sp^2^)-H alkylation of benzaldehyde **(28)** with maleimides **(29)**, with a catalytic amount of aniline as a transient directing group (Li et al., 2018). A variety of pyrrolidines were synthesized with high atom economy and a tolerance for various functional group; only 0.5 mol% of Ru was enough for a 100 mmol scale-up reaction. Forty-six (46) different compounds have been reported. Different substituents that may be electron withdrawing or electron donating at any regio-position, i.e., at ortho-, meta-, or para-positions, were well tolerated, producing moderate to excellent yields (45%–92%). Further, the best yields were obtained when 2-methyl-3-trifluoromethyl aniline was used in the catalytic system as opposed to other anilines and amino acids due to the fact that this ligand affects reversible imine formation (Scheme 3D).

#### 2.1.4 Palladium-catalyzed synthesis

Biswas et al. (2021) developed a synthetic protocol for the synthesis of bridged N-bicyclic heterocycles **(33)** via a palladium-catalyzed reaction starting from γ-C(sp^3^)-H olefination of aminocyclohexane **(31)** with gem-dibromoalkenes **(32)**, followed by copper-mediated intramolecular amidation of 1-bromo-1-alkenylated products (Biswas et al., 2021). Nineteen (19) different compounds have been synthesized by this reaction. On the basis of the substrate structure, 4%–91% yields were obtained. The substituent methyl at position four of cyclohexamines delivered good to excellent yield (trans: 77%, cis: 91%) (Scheme 4A). Deardorff et al., 2020 reported the enantioselective synthesis of the N-containing natural product DAB **36** through an alkene cross-metathesis reaction catalyzed using a combined enzyme and transition metal strategy (Deardorff et al., 2020). The incompatibilities of the substrates with oxynitrilase were overcome via the utilization of a palladium-catalyzed olefine metathesis to functionalize an enzyme-derived (R) allylic fragment (Scheme 4B). Kumar et al. (2018) reported the synthesis of indolines **(38)** starting from anilines **(37)** via an oxidative coupling reaction catalyzed by palladium (Kumar et al., 2018). The indolines were synthesized from the β-amino ketone by intramolecular α-arylation and domino annulation. Further terminal oxidation was achieved by the easily accessible allylic R-OH and O_2_. The importance of this method was the access to β-aryl-ketone compounds as it avoided the use of R-X electrophilic agents and vinyl ketones. Overall, five (5) different compounds have been synthesized by this reaction with moderate to good (37%–62%) yields (Scheme 4C). Cao et al. (2018) reported the synthesis of tricyclic perhydroindoles scaffolds **(40, 41)** via intramolecular transfer hydrogenation and cycloaddition of p-quinamine-tethered alkylidenecyclopropanes (ACPs) **(39)**, mediated by palladium (Cao et al., 2018). Using this method, various polycyclic perhydroindoles derivatives as well as many natural and pharmaceutical products have been synthesized. A total of 15 different perhydroindoles have been synthesized by this methodology. Different alkyl and aryl substituents with electron donating and withdrawing groups were well-tolerated, with moderate to good yields (25%–68%). The best yield (71%) was obtained when 3.0 equivalents of i-PrOH were used with CpPdf (Pi-cinnamyl) at 120°C (Scheme 4D). Chen et al. (2017) described the synthesis of C2-spiropseudoindoxyls **(43)** via a palladium-mediated cycloisomerization/nucleophilic addition/reduction reaction (Chen et al., 2017). This method involved the palladium-mediated cyclization of 5-exo-dig nitroalkyne **(42)** and an internal nitrogen-oxygen bond redox process. A total of 31 different N-heterocycles have been produced by this method. The yields, due to different substrate structures, were moderate to excellent (27%–87%). Using different reaction conditions, good yields (84%) were obtained when a Pd(OAc)_2_ catalyst used with a tmphen ligand, Mo(CO)_6_, reductant, and CF_3_CH_2_OH (TFE) solvent. Among the different solvents used, TFE was good; due to its lower nucleophilic nature it did not react with the carbonyl and it could also lower the coordination degree between the indole-N-oxide and the metal. No reaction occurs when the Mo(C)O)_6_ reductant was replaced by a CO reductant (Scheme 4E). Liu et al. (2021) reported that Ugi-adducts **(44)** can be cyclized intramolecularly with the aid of a cascade dearomatization/azaMichael addition procedure to plicamine derivatives **(45)** containing five-membered saturated pyrrolidine rings using palladium acetate as a catalyst (Liu et al., 2021b). Using a combination of a Ugi-4 component reaction and palladium-catalyzed dearomatization, 14 plicamine analogues were extremely efficiently and cost-effectively produced rapidly. Additional functional group transformations of the keto group via reduction have also been carried out to demonstrate the synthetic value of this approach (Scheme 4F). Using the same strategy, Liu et al. (2021a) reported the synthesis of various zephycarinatine and zephygranditine (bearing pyrrolidine heterocycle) **(45’)** compounds with two adjacent quaternary carbon stereocenters, exhibiting excellent chemoselectivity and stereoselectivity (Liu et al., 2021b). The method employs step-efficient Pd-mediated arylative dearomatization and a subsequent aromatization/dearomatization/aza-Michael addition process of Ugi adducts **(44’)**. This method allows for a wide range of substrates and remarkable functional group tolerance with both electron donating and withdrawing groups (Scheme 4F). Chen et al. (2021) described the synthesis of bicyclic N-heterocycles **(48)** via palladium-mediated intramolecular asymmetric carbopalladation of N-aryl acrylamides **(46)** (Chen et al., 2021). Good yield was obtained when CsOAc was used instead of NaOAc, CsCO_3_, or AcOH because CsOAc acts as a base and as a nucleophile. Good yields were obtained for electron donating groups at para and ortho positions, whereas at the meta position, the yield decreases. Electron withdrawing group were also tolerated at all positions of the aromatic ring and produced a 20%–60% yield. Twenty-one (21) different compounds have been reported by this method. Depending on the structure of R^1^ and R^2^, moderate to excellent (20%–85%) yields were obtained (Scheme 4G).

#### 2.1.5 Copper-catalyzed synthesis

Liu and Buchwald (2020) reported a copper-catalyzed highly regio- and enantioselective polarity-reversed method under mild reaction conditions for the hydroamination of olefins **(49)** to yield a pyrrolidine **(50)** (Liu and Buchwald 2020). The chiral intermediates organocoppers are interdicted by amine electrophilic reagents. The strategy was very scalable because it could be applied to a variety of olefins, including internal olefins. From the synthesis of N-alkylated heterocycles, anilines, amides, and different types of amines, the scope of amine reagents has been extended further. Overall, seven (7) different compounds have been synthesized by this reaction (Scheme 5A). Zhang L. et al. (2020) reported a modern procedure for intramolecular cyclization and intermolecular carbonylative conversion of N-fluoro-sulfonamides **(51)** into N-sulfonyl-β**-**homoproline esters **(53)** in a bipyridine and Cu(OTf)_2_ catalytic system (Zhang T. et al., 2020). Twenty-four different compounds have been synthesized by this reaction. Good yields (71%–79%) were obtained with an increase in the temperature (100°C) and CO pressure (50–60 bar). No desired products were obtained in the absence of a base. Low yields were obtained when LiOH was used as a base, possibly due to the reaction of ester and hydroxide. When the reaction was performed in a mixed solvent system of THF/PhCF_3_ (4:1), a 50% yield of the target product 2a could be obtained (50%–79% yield, Scheme 5B). Hayama et al. (2020) reported a new method for chiral five-membered N-heterocyclic allylboronates **(57)** via Cu(I) the dearomatization of pyrrole **(54)** (Hayama et al., 2020). The reaction involves enantio- and regioselective addition of borylcopper(I) to pyrroles-2-carboxylates, followed by diastereoselective protonation of borylcopper(I) enolate. Five compounds have been synthesized by this reaction. The yield depends on the catalyst, ligand type, and alcohols. When an (S)-MOP ligand was used, no reaction was observed, and the lowest yield (7%) was obtained from the reaction without alcohol additives. The desired product, with excellent enantioselectivity (95%) and yield (81%), was obtained by using a CuCl catalyst in combination with the biphosphine ligand (2R, 4R)-pentane-2,4-diyl[bis(3,5-dimethylphenyl)] phosphine and the base KO^*t*^Bu (Scheme 5C). Wang et al. (2018) described an efficient method for the formation of pyrrolidine-2-carbaldehydes **(59)** from inactivated cyclic amines **(58)** through an oxidative ring contraction via Cu(OAc)_2_/KI/O_2_ (Wang et al., 2018). The strategy required mild reaction conditions, good atom-economy, and easily available substrates. Overall, 22 different compounds were synthesized by this reaction. Moderate yields (51%–59%) were obtained by using the solvent CH_3_CN and the additives KI or I_2_ with a copper catalyst. (Scheme-5D). Rao et al. (2020) reported the synthesis of pyrrolidone **(61)** from the aminooxygention of unactivated alkenes **(60)** with TEMPO (2,2,6,6-tetramethylpiperidin-1-yl)-oxyl using a Cu-catalyst (Rao et al., 2020). Overall, 22 different compounds have been synthesized by this reaction. The desired products were obtained in acceptable to excellent yields (45%–92%) when the solvent MeCN and the bases K_2_CO_3_ or Na_2_CO_3_ were used with Cu(OAc)_2_. Without Cu(OAc)_2_, there was no reaction. The expected products were produced with better yield via the aminooxygenation of unsaturated quaternary amides having bi-phenyl or bi-alkyl substituents at the α-site (Scheme 5E). Wdowik et al. (2020) reported the aerobic enantioselective carboamination of unactivated alkenes **(62)** via a Cu-catalyst for the formation of N-heterocycles **(63)** (Wdowik et al., 2020). Overall, 10 different compounds were synthesized by this reaction. An excellent yield (97%) and high enantioselectivity (92%) were obtained when 10% oxygen in nitrogen was used as an oxidant with 20 mol% of Cu(OTf)_2_. All substituents on the aromatic ring were tolerated and the product was obtained with an excellent yield and high enantioselectivities. CuCl with the chiral ligands (R, R)-Bn-Box (bn = benzyl) and (4S,5R)-bis-Ph-Box, when used in combination, produced good yield but poor or no ee were obtained (Scheme 5F). Deng et al. (2019) reported the synthesis of either exo or endo spirocyclicpyrrolidines **(66 &66’)** through a diastereodivergent method via the asymmetric 1,3-dipolar cycloaddition of azomethane **(64)** ylides with a four-membered ring-containing exocyclic alkenes **(65)** using a Cu catalyst (Deng et al., 2019). This process produced a variety of spirocyclic pyrrolidineazetidine/oxe(thie)tanes with high enantioselectivities (99%) and excellent yields (99%). When iminoester containing electron-rich and electron-deficient groups on the aromatic ring were employed, good yields (78%–91%) with high enantioselectivities (96%–99% ee) and the best diastereoselectivities (13:1-20:1 dr) were obtained (Scheme 5G). Chemler et al. (2017) described the Cu-mediated regio-, stereo-, and diastereoselective synthesis of five-membered N-heterocycles **(68)** via the addition of amine derivatives to un-activated alkenes (Chemler et al., 2017). The substrate (2-Vinylanilines **(67)** and other related substrates) follows allylic amination or alternative aminooxygenation pathways. Both radical and polar steps were involved in the reaction mechanism. The use of CuCl as a catalyst was superior to other copper salts such as Cu(OTf)_2_ and Cu(2-ethylhexanoate)_2_ (Scheme 5H). Kubota et al. documented the formation of chiral indolines **(70)** from the enantioselective borylative dearomatization of indoles **(69)** using chiral bisphosphine-copper(I) and a diboron reagent (Chemler et al., 2017). Thirteen (13) different compounds have been synthesized by this reaction. Different substituents were allowed, and the yields obtained were moderate to outstanding (<5–99%); however, Me-protected indoles were not converted to the expected compounds. Outstanding yields (98%), better enantioselectivities (93% ee), and high diastereoselectivities (97:3 d.r) were obtained under standard reaction conditions, as shown in Scheme 5I.

#### 2.1.6 Iron-catalyzed synthesis

Das et al. (2020) described the formation of complex N-heterocycles **(72)** via Fe-base–mediated enantioselective intramolecular denitrogenative C(sp^3^)-H amination, with high reactivity and functional group tolerance (Das et al., 2020). Overall, 32 different compounds have been synthesized by this reaction. The desired products were obtained in moderate to excellent yields (18%–98%). Excellent yields (80%–100%) were obtained when 10 mol% of Fe(TPP)Cl-iron(tetraphenylporphyrinato) chloride was used with 20 mol% of Zn (reductant) and benzene as the solvent at 120–150°C. High yields were also obtained with substrates having primary, secondary, and tertiary C-H bonds and various substituents at different positions (Scheme 6A). Cornil et al. (2015) reported diverse pyrrolidine **(74)** synthesis from σ-amino allylic acetate derivatives **(73)**, mediated by FeCl_3_.6H_2_O (Cornil et al., 2015). This strategy is important for the synthesis of a variety of natural products. The desired products were obtained with moderate to excellent diastereoselectivity and excellent yields (88%–99%). Overall, three (3) different compounds have been synthesized by this reaction (Scheme 6B). Bagh et al. (2017) reported the formation of different saturated N-heterocycles **(77)** via the intramolecular C(sp^3^)-H amination of unactivated organic azides **(75)**, mediated by an air stable Fe(III) species with a redox active ligand (Bagh et al., 2017). The method employs an Fe(III) catalyst that was readily recycled without any loss of catalytic activity. Eight (8) different compounds have been synthesized by this reaction. The yields, due to different substrates, were moderate to excellent (39%–96%). The use of excess Boc_2_O did not lead to any useful change in the desired product quantities; however, when the concentration of the catalyst was increased up to 5 mol%, the full conversion of substrates into the desired products occurred after 3 h. When 0.1 mol% of catalyst was loaded, only 11% or 23% of the desired products was obtained (Scheme 6C). Henry et al. (2019) reported the synthesis of indolines **(80)** via iron and copper catalysts in an easy one pot two step intramolecular aryl C-N bond forming process (Henry et al., 2019). This one pot strategy was applied for the synthesis of different indolines, dihydrobenzofurans, neolignan natural products, (+)-obtusafuran, and other six-membered analogues. A total of 16 different compounds were synthesized by this reaction. A range of N-tosyl ethylamine, substituted anisoles, acetanilides, and anilines were converted to indolines, and the yields obtained were moderate to excellent (43%–93%). Phenylethylamine having N-acetyl and chlorine substituents was not converted by an iron (III) catalyst even at 70°C (Scheme 6D).

#### 2.1.7 Nickel-catalyzed synthesis

Dong R. et al (2020) described a good method of C-H amination for the formation of pyrrolidines **(82)** from an aliphatic azide substrate **(81)** catalyzed by a Ni-dipyrrinato complex (Dong Y. et al., 2020). This process allowed for mild reaction conditions, low catalyst loading, a wide scope of substrates, and high chemoselectivity. Overall, 28 compounds have been synthesized by this reaction. The yield was excellent (94–>99%) when 1–10 mol% of catalyst (^AdF^L)Ni(py) was used with C_6_D_6_, hexane, or THF solvents and a time of 10–90 min. The yield did not vary significantly with the change of solvent polarity, e.g., from hexane to THF. No yield was observed when DMSO and MeOH were used as the solvent, most likely due to decomposition of the catalyst by demetallization. When dichloromethane (DCM) was used as the solvent, no yield was observed because a divalent nickel-chloride complex was formed. Different substituents at different positions were also well-tolerated and moderate to excellent yield was observed (Scheme 7A). Sardini et al. (2019) reported the synthesis of diverse, pharmaceutically important pyrrolidines **(84)** from the readily available unsaturated nitrogen heterocycles **(83)** using a Ni catalyst (Sardini et al., 2019). The desired products were synthesized with controlled regio-, stereo-, and diastereoselectivity, and was synthetically important. A total of four (4) different saturated heterocycles were synthesized with good to excellent (64%–95%) yields via transformation of the Bpin unit from the parent compound. The scope of this specific reaction can be enhanced by varying the aryl group attached (Scheme 7B). Zheng et al. (2019) described the synthesis of pyrrolidinone core–containing complex molecules **(87)** from the amidoarylation of unactivated olefins **(85)** by a visible light photocatalyst and a nickel catalyst (Zheng et al., 2019). Different substrates were well-tolerated and a large number of saturated N-heterocycles have been synthesized using this photoredox PCET/Nickel dual catalysis method (Scheme 7C). Liu et al. (2018) described the preparation of pyrrolidines **(89)** from the highly enantioselective intramolecular reductive cyclization of N-alkynones **(88)** (Liu et al., 2018). The reaction used Ni with the P-chiral biphosphorus ligand DI-BIDIME and triethylsilane as a reducing agent. Overall, 22 compounds have been synthesized by this reaction. Substrates with different N-protecting groups showed various reactivity with moderate to excellent yields (10%–98%). Substrates with N-Bn and N-Tf groups reacted slowly with lower yields (10%–19%). Substrates with different electron donating, electron withdrawing, and indole moieties were well-tolerated with good to excellent yields obtained (76%–98%). When the N-Boc group was employed as the N-protecting group, no conversion takes place (0% yield). The desired compounds were obtained with good yields and enantioselectivities in the presence of 0.1 mol% of Ni(cod)_2_ and 0.05 mol% of (S, S)-DI-BI-DIME, with 2 mol of substrate at 60°C for 12 h (Scheme 7D).

#### 2.1.8 Cobalt-catalyzed synthesis

Baek and Betley (2019) developed the synthesis of substituted pyrrolidines **(91)** from the C-H amination of alkyl azides **(90)** via dipyrrin-supported Co^III^ imidos (Baek and Betley 2019). The presence of pyridine (cocatalyst) plays a key role because pyridine coordination inhibited the formation of a four-coordinated Co-tetrazido complex, which was catalytically unactive; it also promoted C-H amination under milder conditions. This method showed that transition metal ligands with multiple bonds can be used to prepare new catalysts. Overall, four (4) different compounds have been prepared by this reaction. The yield increased to 93% with an increase in the concentration of pyridine; however, without pyridine, the yield was very low (<10%) because of the lower C-H amination rate (scheme-36). Herbort et al. (2021) reported the synthesis of pyrrolidines **(93)** via the intermolecular hydroalkenylation of 1,6-enynes **(92)**, catalyzed by cationic bis-diphenylphosphinopropane (DPPP)Co^I^ (Herbort et al., 2021). In this reaction, an intermediate cobaltacyclopentene was formed via the oxidative cyclization of enyne, which, upon reaction with alkene, produces regiodivergent products. The use of the ligand 1,3-bis-diphenylphosphinopropane (DPPP) instead of 1, n-bis-diphenylphosphino-alkanes, as well as the chlorinated solvents dichloromethane (DCM) and 1,2-dichloroethane (DCE) instead of toluene and diethyl ether delivered good yield. Twelve different compounds have been synthesized by this reaction (scheme-37).

#### 2.1.9 Titanium-palladium (mixed)–catalyzed synthesis

Ence et al. (2019) described the synthesis of pyrrolidine **(95)** catalyzed by a Ti-Pd complex via the intramolecular allylic amination of an N-alkyl electrophile **(94)** (hindered substrate) within less than 10 min at room temperature (Ence et al., 2019). Moderate enantioselectivities and yields were observed with chiral ligands that lack titanium. The enantioselectivity increased with an increase in the reaction time and also with the introduction of ortho-tolyl substituents on aryl phosphine moieties of the different ligands to form complexes. The increased enantioselectivity after full consumption of the substrate suggested a reversible enantio-determining step that enables the equilibration of product enantioselectivity via a kinetic resolution type method. The substrate structure also plays a key role because, under the same reaction conditions, pyrrolidines were obtained with lower enantioselectivity as compared to piperidine (Scheme 9).

#### 2.1.10 Gold- and iridium-catalyzed synthesis

Liu et al. (2022) reported the highly efficient economical novel synthesis of quinazolinone and ampakine analogues **(97’)** mediated by the Au(I) cascade intramolecular cyclization of Ugi adducts **(96’)** (Liu et al., 2022). Good yields, great functional group tolerance, a wide range of substrates, and outstanding regio- and chemoselectivity are all characteristics of this technique. An additional example of this strategy’s viability is its scale-up potential. It is also discovered that different oligopeptides are well-tolerated, allowing for polycyclic products that are enriched with peptide chains (Scheme-10A). Ohmura et al. (2020) reported the conversion of o-alkyl-N-methylanilines **(96)** to indolines **(97)** through C-H transformations catalyzed by iridium with a DTBM-SEGPHOS ligand and tert-butylethylene as a hydrogen scavenger (Ohmura et al., 2020). Using this strategy, indolines having a quaternary stereogenic carbon center at the C3 position have been synthesized via enantioselective dehydrogenation-intramolecular C-H addition catalyzed by iridium. Overall, six (6) different compounds have been synthesized by this reaction. The products were obtained with excellent yields (77%–93%) and high enantioselectivity (77%–94%) (Scheme 10A). Wu et al. (2016) reported the synthesis of chiral five-membered aza-spiroindolenines **(99)** via the iridium**-**catalyzed allylic dearomatization reaction of indoles **(98)** (Wu et al., 2016). The pictet-spingler type of compounds were synthesized using this process, with an additional allylic stereogenic center adjacent to the C3 position of indole via an asymmetric dearomatization reaction under enhanced reaction conditions. Overall, 15 different compounds have been synthesized by this reaction (Scheme 10B). Kotha and Sreevani (2018) reported [2 + 2+2] cyclotrimerization for the synthesis of isoindolinone-based (halomethyl)benzenes **(102)** from propargyl halides **(101)** and unsymmetrical diyne **(100)** using a Mo(CO)_6_ catalyst (Kotha and Sreevani 2018). Overall, 10 different five-membered N-heterocycles have been synthesized by this method. The yields were good to moderate (48%–99%) (Scheme 10C).

### 2.2 O-HETEROCYCLES

#### 2.2.1 Rhodium-catalyzed synthesis

Wei et al. (2021) reported the synthesis of Rh(III)-mediated THF **(105)** through the stereo/regioselective C-H coupling/C-terminus Michael addition reaction of N-phenoxy amides **(103)** with 1,6-enynes **(104)** (Wei et al., 2021). Under optimal reaction conditions, a series of N-phenoxyacetamides **(103)** having different functional groups and a variety of 1,6 enynes **(104)** having alkyl or aryl groups at position 4 gave moderate to good yields (34%–73%). Good to moderate yields were obtained by using an alcoholic solvent, especially TFE with a Zn(OAc)_2_ base and a Rh(III) catalyst. By using Ru(II) catalysts instead of Rh(III), no yield was obtained. Overall, 25 compounds have been synthesized by this reaction (Scheme 11A). Lindsay et al. (2015) described the synthesis of 2,3-disubstituted tetrahydrofurans **(107)** catalyzed by Rh(II) via intramolecular azavinyl carbene C(sp^3^)-H insertion, with excellent distereoselectivity from easily available acyclic substrates **(106)** (Lindsay et al., 2015). Fused N-heterocycles could also be directly prepared in one step using this intramolecular C-H amination. Overall, 13 different compounds have been synthesized by this reaction. The yield ranged from 5% to 96%. The catalyst Rh_2_(tpa)_4_ gave optimal diastereoselectivity; however, the use of CH_2_Cl_2_ solvent led to an increase in both the yield and diastereoselectivity. The yield and diastereoselectivity, along with the catalyst and solvent nature also depended on the time. As the time increased from 1 h to 4.5 h, the yield increased from 23% to 96% and the diastereoselectivity from 38:62 dr to 96:4 dr (Scheme 11B).

#### 2.2.2 Palladium-catalyzed synthesis

Verdugo et al. (2020) documented the formation of highly substituted tetrahydrofurans **(110)** via (3 + 2) heterocycloadditions between alkylidenecyclopropanes **(108)** and carbonyls **(109)** catalyzed by a palladium catalyst (Verdugo et al., 2020). The importance of this strategy was that the annulation allows a direct approach to obtain fused polycyclic systems with β-methylene tetrahydrofuran moieties. The Pd(0) catalyst mediated the cycloadditions between trifluoromethyl-acetophenones and ACPs, allowing chiral THFs with trifluoromethyl-substituted carbons. It is also notable that no reaction occurred with aliphatic ketones. Overall, six (6) different compounds have been synthesized by this reaction. Different substituents were well-tolerated and the yield was excellent (40%–95%, Scheme 12A). Ye et al. (2020) reported the synthesis of spiro-fused O-heterocycles **(113)** with good chemo-, stereo-, and regioselectivity from allyl ammonium salts **(112)** and aryl halides **(111)** catalyzed by palladium (Ye et al., 2020). Using this method, 48 spiro-fused dihydrobenzofuranes and indolines **(113)** with various fluorinated derivatives have been synthesized. Overall, 45 different spiro-fused O-heterocycles have been synthesized by this reaction. The yields, due to different substates, were moderate to excellent (40%–94%). No desired products were obtained when 2-phenylallyl acetate and tert-butyl (2-Phenylallyl) carbonate were used as substrates (Scheme 12B). Yu et al. (2020) described the synthesis of various spirofuran-hydropyrimidinones **(117)** via palladium and Bronsted acid co-catalysts via the reaction of alkynol **(116)** with (thio)urea **(115)** and aromatic aldehydes **(114)** (Yu et al., 2020). Using this method, a variety of O- and N-heterocycles have been prepared with good yield and high enantioselectivity from various substrates and with sensitive functional group tolerance. Overall, 25 different compounds have been synthesized by this reaction. The yields, due to various substrates, were moderate to excellent (45%–87%). In this method, various aromatic aldehydes were transformed into the desired products. Using benzaldehyde with electron donating substituents and with substituents at the para position produced good yields as compared to those with electron withdrawing substituents and substituents at the meta position; however, ortho-substituted benzaldehydes required a longer time for the full conversion of reactants. Using 20 mol% of TFA, 10 mol% of PdCl_2_, and 1,4-dioxane as a solvent, excellent yields (86%) were obtained (Scheme 12C).

#### 2.2.3 Copper-catalyzed synthesis

Wdowik et al. (2020) reported the preparation of spirocyclic ethers **(119)** via the enantioselective aerobic intramolecular carboetherification of unactivated alkenes **(118)** using a copper catalyst (Wdowik et al., 2020). Overall, nine (9) different compounds have been synthesized by this reaction. The yield was dependent upon the nature of the catalyst, type of ligands, oxidant, and additives. The use of Cu(OTf)_2_ mixed with 10% O_2_ in N_2_ in the presence of the ligand but without additive gave very good yields (81%) and enantioselectivity (86%). Good yields were also observed when CuCl was used with AgOTf and ligand (Scheme 13A). Xie et al. (2017) reported the aminooxygenation of alkenes **(120)** for the synthesis of aminated saturated O-heterocycles **(121)** catalyzed by copper (Xie et al., 2017). This method produced a variety of aminated saturated O-heterocycles with good yields and required mild conditions, was simple, and allowed the use of a wide variety of substrates. The aminooxygenation of internal alkenes produced O-heterocycles with excellent diastereoselectivity. Overall, 16 different compounds have been synthesized by this reaction. Good yields were obtained by using CuCl and DCE as a solvent instead of DMF, MeCN, or 1,4-dioxane. The reaction occurred at room temperature. Different substituents were well-tolerated (Scheme 13B). He et al. (2015) described the synthesis of saturated O-heterocycles **(124)** from allylic alkynoates **(123)** using a copper catalyst. This method was used for the preparation of different heterocyclic compounds of O- and N-heteroatoms as well as biologically active CF_3_-containing compounds (He et al., 2015). Overall, seven different compounds have been synthesized by this reaction. The yields were moderate to good (45%–69%) (Scheme 13C).

#### 2.2.4 Iron-catalyzed synthesis

Henry et al. (2019) reported the synthesis of dihydrobenzofurans **(126)** via an iron-catalyzed one-pot two-step intramolecular aryl C-O bond forming process using copper as a co-catalyst (Henry et al., 2019). A total of nine (9) different compounds have been synthesized by this reaction. The yields were moderate to good (29%–72%) (Scheme 14A). Watile et al. (2019) reported the synthesis of saturated nitrogen, oxygen, and sulfur heterocycles via the intramolecular substitutions of secondary and tertiary alcohols, with chirality transfer promoted by an iron(III) catalyst (Watile et al., 2019). Two different compounds of O-heterocycles **(128)** have been synthesized by this reaction. The yields were excellent (99%) (Scheme 14B).

#### 2.2.5 Cobalt-catalyzed synthesis

Shigehisa et al. (2016) described the synthesis of substituted THF **(131)** via hydroxylation of unactivated alkenes **(130)** through “unprotected” and “protected” strategies catalyzed by cobalt (Shigehisa et al., 2016). The catalyst system was developed using a cobalt salen complex, N-fluoro-2,4,6-trimethylpyridinium salt, and (Me_2_SiH)_2_O. Four different substituted THFs have been prepared by this method; however, in Scheme-15A, only one specific example is represented for the sake of simplicity. Different substituents were tolerated and a variety of O-heterocycles were synthesized by this reaction with moderate to excellent yields (13%–97%). Furthermore, seven different starting materials with different protective groups were used for the synthesis of the specific example shown in Scheme 15A to investigate the scope of the protective group. When the protective groups for the hydroacyloxylation of unactivated olifines were Me, TBS, MOM, and BOM, 99% yield was obtained within 0.5 h; however, when protecting group was Ac, 27% yield was obtained (Scheme 15A). Gandeepan and Cheng. (2015) developed the cobalt-catalyzed synthesis of a variety of saturated O-heterocycles **(133)** via the intramolecular reductive coupling of alkynes **(132)** with activated alkenes (Gandeepan and Cheng 2015). In this readily available method, an environmentally friendly and less toxic cobalt catalyst was used for many kinds of reactions such as addition, cycloaddition, reductive coupling, carbocyclization, C-H activation, and cross coupling. Overall, 25 different compounds have been synthesized by this reaction. The yields were moderate to excellent (40%–94%, Scheme 15B). Ebisawa et al. (2020) described the synthesis of tetrahydrofurans **(135)** via a radical polar crossover (RPC) and transition metal–catalyzed hydrogen atom transfer (TM-HAT) mechanism (Ebisawa et al., 2020). A variety of chiral tetrahydrofurans have been synthesized with high enantioselectivity (up to 94% ee) and absolute configurations by using a chiral cobalt catalyst, N-fluoro-2,4,6-collidinium tetrafluoroborate, and diethylsilane (cobalt salen complex; very similar as that shown in scheme 15a). Overall, 22 different compounds have been synthesized by this reaction. The yields obtained, due to different substrates, were moderate to good (12%–84%) and the enatioselectivities were moderate to excellent (6%–94% ee, Scheme 15C).

#### 2.2.6 Titanium-catalyzed synthesis

**Zhao et al** documented a new methodology for the preparation of chiral tetrahydrofuran derivatives **(137)** via the asymmetric intramolecular hydroxylation of unactivated alkenes **(136)** mediated by TiCl_4_ and chiral N-triflyl phosphoramide (Wang W. et al., 2020). Although it has moderate enatioselectivity, the strategy allows the asymmetric hydroxylation of challenging unactivated alkenes. Eleven (11) different compounds have been synthesized by this reaction. The yields of tetrahydrofuran derivatives were moderate to excellent (51%–99%) with excellent enantioselectivities (30%–71%). No yields (0%) were observed when Ti(O^i^Pr)_4_ or HCl were used instead of TiCl_4_; even the chiral N-triflyl phosphoramide could not promote the reaction without TiCl_4_ (Scheme 16).

#### 2.2.7 Gold-catalyzed synthesis

Garre et al. (2019) described the gold-catalyzed preparation of cyclobutane-fused THF **(139)** via regiodivergent electrophilic cyclyzation of alkynylcyclobutanes **(138)** (Garre et al., 2019). Using this strategy, a variety of cyclobutane-fused O-heterocycles found in various natural products have been prepared. Sixteen (16) different compounds have been synthesized by this reaction. Different substituents were tolerated and moderate to excellent yields (12%-99%) were obtained (Scheme 17A **)**. Zhang Y. et al (2020) developed the synthesis of saturated benzo-fused THF **(141)** using visible light and radical carbo-cyclization/gem-diborylation via triplet energy transfer between aryl iodide and a gold catalyst (Zhang L. et al., 2020). Using this method, an important polymer, two approved drugs, indole-, benzofuran-, and benzothiophene-based benzylic gem-diboronates have been produced with high functional group tolerance (Scheme 17B).

### 2.3 S-HETEROCYCLES

#### 2.3.1 Iron-catalyzed synthesis

Watile et al (2019) reported the synthesis of saturated nitrogen, oxygen, and sulfur heterocycles via the intramolecular substitutions of secondary and tertiary alcohols **(142)**, with chirality transfer promoted via an iron(III) catalyst (Watile et al., 2019). This method required an easily available and inexpensive iron-catalyst for the synthesis of important five-membered, six-membered, and aryl-fused six-membered heterocyclic compounds containing O-, S-, and N-heteroatoms. Two different compounds of S-heterocycles **(143)** have been synthesized by this reaction. The yields were excellent (91%–98%) (Scheme 18A).

#### 2.3.2 Cobalt-catalyzed synthesis

Date et al. (2020) reported the preparation of saturated sulfur-heterocycles **(146)** from alkenyl thioester, present in the structure of many biological molecules and pharmaceuticals (Date et al., 2020). The catalytic system used was a cobalt (salen) complex, which was used in Scheme 15A,C. The reaction was mediated by a cobalt hydride hydrogen atom transfer (HAT) and radical polar crossover (RPC) mechanism, enabling simultaneous cyclization and deprotection. Under optimized conditions, this strategy was applicable to different substrates. A total of 22 different compounds have been synthesized by this reaction. Different substrates were well-tolerated and excellent yields were obtained (16%–94%, Scheme 18B **)**.

## 3 Saturated heterocycles containing two heteroatoms

### 3.1 Ruthenium-catalyzed synthesis

Zhou et al. (2020) described the synthesis of chiral 2-imidazolidines **(148)** with excellent yields (up to 99%) and enantioselectivities (up to 99%) via the enantioselective intramolecular C(sp_3_)-H amination of N-benzoyloxyurea **(147)** mediated by ruthenium (Zhou et al., 2020). The catalyst used was a chiral metal ruthenium complex in which two bidentate N-(2-pyridyl)-substituted N-heterocyclic carbenes and two acetonitrile ligands are coordinated to a central ruthenium atom in a C_2_-symmetric fashion. A total of 24 different compounds have been synthesized by this reaction. The yields obtained were moderate to excellent (29%–99%). The enantioselectivities were also moderate to excellent (40%–99.9%). Different substituents, such as ethyl, n-butyl, isobutyl, and phenethyl, were well-tolerated and the desired products were obtained with 68%–99% yields with 92%–95% ee; however, a moderate yield (37%) was obtained from benzyl substituents (Scheme 19A). Using the same catalyst system, i.e., a chiral metal ruthenium complex, the same research group demonstrated a process for the preparation of chiral imidazolidine-4-ones **(150)** via the enantioselective ring-closing C-H amination of 2-azidoacetamides **(149)** using a ruthenium catalyst (Zhou Y. et al., 2019). In this method, the highly enantioselective C(sp^3^)-H amination of aliphatic azides takes place, producing useful biological substances, amino acids, and asymmetric organocatalysts. A total of 23 different compounds have been synthesized by this process. The yields were moderate to excellent (31%–95%). The enantioselectivities, due to different substrates, were also moderate to excellent (23%–95% ee) (Scheme 19B).

### 3.2 Copper-catalyzed synthesis

Zhan et al. documented the preparation of trifluoromethylated 2-oxazolidone **(153)** from the combination of CO_2_ Togni’s reagent and a variety of allylamines **(151)**, catalyzed by Cu-TDPAT (Zhang L. et al., 2020). Using this strategy, biologically important oxazolidinones and 2-thiazolidinethiones have been synthesized by employing a highly durable Cu(II) melamine coordination polymer, Cu-TDPAT, for the heterogeneous photocatalytic oxytrifluoromethylation of allylamine and carbon dioxide. A total of seven (7) different compounds have been synthesized by this reaction. The yields, due to different substrates, were good (60%–75%). Improved yields (77%) were obtained when 5 mol% of Cu-TDPAT was used in combination with Xe light (>400 nm), the base DBU, and the solvent MeCN (Scheme 19C). Chen et al. (2020) reported the synthesis selenyl 2-oxazolidinones **(156)** via the electrophilic oxyselenation of propargylic amines **(154)** with diselenides **(155)** and carbon dioxide at atmospheric pressure, catalyzed by Cu/DTBP (Chen et al., 2020). This strategy required mild reaction conditions and was applicable to various substrates; selenyl 2-oxazolidinones, useful in biological fields, have been obtained with good yields. This process could be applied to terminal and non-terminal propargylic amines. Overall, 43 different compounds have been produced by this method. The yields due to terminal propargylic amines were good to excellent (75%–92%). The yields due to nonterminal propargylic amines were low to excellent (0%–90%, Scheme 19D). Dong R. et al. (2020) reported the synthesis of fused heterocyclic polymers, with imidazo [2,1-b] thiazolels **(160)** units in their main skeleton, via a multicomponent one pot polymerization reaction using a copper catalyst (Dong Y. et al., 2020). Under optimized reaction conditions, this strategy produced a variety of fused heterocyclic polymers that contain imidazo [2,1-b] thiazole **(160)** units, with high molecular weight and moderate yields obtained (up to 54.3%). The polymers produced by this method possess small energy bands, excellent thermal stability, and excellent solubility. DMSO was found to be the best solvent. The yields were 15%–54.3% when DMSO at 110°C or 80°C and 0.2 equiv. of copper salts were used. Using solvents other than DMSO, such as THF, DCM, or CH_3_CN, obtained no products (Scheme 19E).

### 3.3 Iron-catalyzed synthesis

Zhong et al. (2019) reported the synthesis of sultams **(162)** and cyclic N-sulfonyl ketimines **(163)** based on the iron complex catalyst Fe(ClO_4_)_2_ and amino pyridine ligand intramolecular aliphatic C(sp^3^)-H amidation (Zhong et al., 2019). Using this strategy, sultams (up to 89%) and N-sufonyl ketimines (up to 92%) were synthesized with good yields; the reaction required an easily obtainable catalyst and was applicable to various substrates. Overall, 22 different sultam compounds have been prepared by this method. Different substrates were well-tolerated and moderate to excellent yields (50%–88%) were obtained. Excellent yields (90%) were obtained when 20 mol% of ligands were used with a PhI(OPiv)_2_ oxidant; however, no yields were obtained without ligands (Scheme 19F).

### 3.4 Nickel-catalyzed synthesis

Liu et al. (2020) explored the synthesis of chiral 2-oxazolidinones **(165)** via asymmetric hydrogenation of 2- oxazolones **(164)** using a nickel catalyst (Liu et al., 2020). The desired products were obtained with excellent enantioselectivities (97%–>99%) and yields (95%–99%). This process required low loading of the catalyst and the chiral 2-oxazolidinones were further transformed into chiral dihydrothiophene-2(3H)-thione, amino alcohols, oxazoline ligand, and other valuable molecules, without the loss of ee or yield. A total of 24 different compounds have been synthesized by this reaction. The yields were moderate to excellent. Under standard reaction conditions, 4,5-disubstitutes 2-oxazolone and 3-oxazolone 5-phenyloxazol-2(3H)-one did not hydrogenate under this catalytic procedure (Scheme 19G).

### 3.5 Cobalt-catalyzed synthesis

Lee et al. (2020) described the synthesis of cyclic carbamates **(167)** via the intramolecular C-H nitrene insertion of azidoformates **(166)** mediated by a Co-catalyst system of Cp*Co(III) (LX) (Lee et al., 2020). The ligand used in the catalyst system was 5,7-dichloro-8-hydroxy-quinoline, while X was bromide and chloride. The cobalt complexes were easily synthesized, required milder reaction conditions, and were used for both C(sp^2^)-H and C(sp^3^)-H carbamation reactions. Overall, 33 different compounds have been synthesized by this reaction. The yields, due to C(sp^2^)-H amidation of phenyl azidoformates, were moderate to excellent (43%–99%). The yields, due to C(sp^3^)-H amidation of azidoformates, were <5–99% (Scheme 20A). Pan et al. (2020) described a three-component Co-catalyzed selectivity controllable radical coupling reaction for the synthesis of imidazoline derivatives **(171)** from the readily available p-anisidine **(169)**, carbonyl compounds **(168)**, and N-phenyl glycine **(170)** in photoredox conditions (Pan et al., 2020). The reaction required mild conditions and different functional group were well-tolerated, which are favorable for diversity oriented synthesis (DOS) in the discovery of drugs and also for the synthesis of mainserine skeleton. Overall, 29 different compounds have been synthesized by this reaction. Different substrates were well-tolerated with good yields obtained (28%–87%) (Scheme 20B). Hu et al. (2019) described the cobalt-catalyzed synthesis of five-membered cyclic sulfonamides **(173)** with good yields with enantioselectivities from both alkylsulfonyl and arylsulfonyl azides **(172)** via radical 1,5-C-H amination (Hu et al., 2019). This method provided the most common and selective catalytic system for the asymmetric C-H amination of sulfonyl azides for the synthesis of five-membered cyclic sulfonamides **(173)** under neutral and nonoxidative conditions. A total of 25 different compounds have been synthesized by this reaction. The yields and enantioselectivity depend on the nature of the cobalt catalyst, type of solvent, and temperature. The lowest enantioselectivity (28% ee) was obtained when a [Co(P1)], P1 = 3,5-Di^*t*^Bu-ChenPhyrin catalyst was used with chlorobenzene as a solvent at 80°C, whereas the highest enantioselectivity (92% ee) was obtained when a [Co(P4)], P4 = 2,6-DiMeOZhuPhyrin catalyst was used with chloroform as a solvent at 50°C. The yields were good to excellent (63%–99%) due to different reaction conditions, i.e., Co catalysts, solvents, and temperature. The yields, due to different substrates, were also good to excellent (62%–99%) and the enantioselectivities moderate to excellent (7%–99% ee, Scheme 20C **)**.

### 3.6 Iridium-catalyzed synthesis

**Bode et al** reported the synthesis of a variety of silapyrrolidines **(176)** from aliphatic amines **(175)** via β-selective C(sp^3^)-H silylation mediated by an iridium catalyst (Jindakun et al., 2018). The reactions occurred at a faster rate, with high enantioselectivity, and at or even below room temperature when promoted by chiral pyridyl imidazoline ligands. In total, 21 different compounds have been synthesized by this reaction. Different substrates were allowed, and the yields were moderate to excellent (37%–84%). The yields depended on the presence or absence of ligands and also on the type of ligands. In the absence of any chiral ligands, 70% conversion takes place, and the yield was only 6%. When different ligands were used, >99% conversion takes place, and the yields were moderate to excellent (46%–91%) (Scheme 20D).

### 3.7 Gold-catalyzed synthesis

Bayrakdar et al. (2020) reported the synthesis of five-membered nitrogen and oxygen-containing saturated heterocycles **(179)** via the carboxylative cyclization of propargylamine (PPA) **(177)** using dinucearl gold(I) complexes with alkyl-bridged bis(N-heterocyclic carbene) ligand as the catalyst (Bayrakdar et al., 2020). Using this method, various new dinuclear gold(I) complexes with bridged bis(NHC) ligands (used as a catalyst for carboxylative cyclization of PPA) have been produced in the presence of air and under moderate reaction conditions. Excellent yields (up to 99%) were obtained at room temperature in the presence of the catalyst and MeOH solvent as well as a reaction time of 24 h. The yields, due to different reactions conditions, were 4%–99% (Scheme 20E).

## 4 Synthesis of five-membered saturated heterocycles with 3-heteroatoms

### 4.1 Palladium-catalyzed synthesis

Garlets et al. (2016) described the synthesis of cyclic sulfamides **(182)** via alkene carboamination between aryl or alkenyl bromides **(181)** and N-allylsulfamides **(180)** using an asymmetric enantioselective palladium catalyst with good enantioselectivities and yields obtained (Garlets et al., 2016). The importance of this strategy was that the reaction occurred via the syn-aminopalladation of alkenes, as indicated by deuterium labelling; this syn- and anti-aminopalladation course was important for asymmetric induction. A total of 27 different compounds have been synthesized by this reaction. Different substrates were tolerated and good yields and diastereoselectivities were achieved (Scheme-20F).

### 4.2 Cobalt-catalyzed synthesis

Lu et al. (2016) developed the synthesis of five-membered cyclic sulfamides **(184)** via the intramolecular 1, 5-C(sp^3^)-H amination of sulfamoyl azides **(183)** under neutral conditions catalyzed by Co(II) (Lu et al., 2016). Using this method, a variety of C(sp^3^)-H bond aminations take place, with good chemoselectivities toward allylic and propargylic C-H bonds. Different functional groups were well tolerated. A total of 20 different compounds have been synthesized by this reaction. The yields obtained, due to different substrate structures, were moderate to outstanding (50%–99%) (Scheme 20G).

## 5 Conclusion

In this mini review, a broad spectrum of novel and efficient transition metal–catalyzed syntheses of saturated five-membered N-, O-, and S-heterocycles from the recent past have been summarized. Clearly, much work has been done in this direction, employing various types of reactions, including cycloaddition, metathesis, coupling, asymmetric reactions, etc. Rhodium, ruthenium, palladium, cobalt, nickel, copper, and iron are the most frequently used transition metals, while gold and iridium have also been used. Heterocycles have been synthesized with high enantioselectivity, regioselectivity, and diastereoselectivity. The commonly modified synthetic methods have improved synthetic research on the transition metal–catalyzed synthesis of saturated heterocycles, with a trend toward milder, effective, convenient, faster, diversified, and higher yielding approaches. In addition, due to their simplicity and cost-effectiveness, single-pot synthetic methods enable the construction of the main scaffolds of different marketed drugs; therefore, it is hoped that further research on designing novel functionalized and diversified saturated heterocycles, exploiting their medicinal activities and pharmaceutical activities, will be carried out. This review highlights the current deviation of interest toward saturated heterocycles; their poor commercial availability enhances the urge for synthetic methods offering diversity and using easily available starting materials. Regardless of the considerable attempts reviewed, a very limited number of methods have provided facile synthesis of a large variety of saturated heterocycles. In the majority of reactions, nitrogen protective groups are required, which are difficult to remove, thereby decreasing the yields and economy. Although few transition metal–catalyzed approaches are very effective, they are restricted to the synthesis of pyrrolidines.
